# Clinical diagnoses associated with a positive antinuclear antibody test in patients with and without autoimmune disease

**DOI:** 10.1186/s41927-023-00349-4

**Published:** 2023-08-07

**Authors:** Jacy T. Zanussi, Juan Zhao, Wei-Qi Wei, Gul Karakoc, Cecilia P. Chung, QiPing Feng, Nancy J. Olsen, C. Michael Stein, Vivian K. Kawai

**Affiliations:** 1 Division of Clinical Pharmacology, Department of Medicine, Vanderbilt University Medical Center, Nashville, TN USA; 2 Department of Biomedical Informatics, Vanderbilt University School of Medicine, Nashville, TN USA; 3 Vanderbilt Genetics Institute, Vanderbilt University School of Medicine, Nashville, TN USA; 4 Division of Rheumatology, Department of Medicine, Vanderbilt University Medical Center, Nashville, TN USA; 5 Tennessee Valley Healthcare System - Nashville Campus, Nashville, TN USA; 6 Department of Medicine, Penn State Milton S. Hershey Medical Center, Hershey, PA USA

**Keywords:** Antinuclear antibodies, PheWAS, Disease risk

## Abstract

**Background:**

Antinuclear antibodies (ANA) are antibodies present in several autoimmune disorders. However, a large proportion of the general population (20%) also have a positive test; very few of these individuals will develop an autoimmune disease, and the clinical impact of a positive ANA in them is not known. Thus, we test the hypothesis that ANA + test reflects a state of immune dysregulation that alters risk for some clinical disorders in individuals without an autoimmune disease.

**Methods:**

We performed high throughput association analyses in a case–control study using real world data from the de-identified electronic health record (EHR) system from Vanderbilt University Medical Center.

The study population included individuals with an ANA titer ≥ 1:80 at any time (ANA +) and those with negative results (ANA-). The cohort was stratified into sub-cohorts of individuals with and without an autoimmune disease. A phenome-wide association study (PheWAS) adjusted by sex, year of birth, race, and length of follow-up was performed in the study cohort and in the sub-cohorts. As secondary analyses, only clinical diagnoses after ANA testing were included in the analyses.

**Results:**

The cohort included 70,043 individuals: 49,546 without and 20,497 with an autoimmune disease, 26,579 were ANA + and 43,464 ANA-. In the study cohort and the sub-cohort with autoimmune disease, ANA + was associated (*P* ≤ 5 × 10^–5^) with 88 and 136 clinical diagnoses respectively, including lupus (OR ≥ 5.4, *P* ≤ 7.8 × 10^–202^) and other autoimmune diseases and complications. In the sub-cohort without autoimmune diseases, ANA + was associated with increased risk of Raynaud’s syndrome (OR ≥ 2.1) and alveolar/perialveolar-related pneumopathies (OR ≥ 1.4) and decreased risk of hepatitis C, tobacco use disorders, mood disorders, convulsions, fever of unknown origin, and substance abuse disorders (OR ≤ 0.8). Analyses including only diagnoses after ANA testing yielded similar results.

**Conclusion:**

A positive ANA test, in addition to known associations with autoimmune diseases, Raynaud’s phenomenon, and idiopathic fibrosing alveolitis related disorders, is associated with decreased prevalence of several non-autoimmune diseases.

**Supplementary Information:**

The online version contains supplementary material available at 10.1186/s41927-023-00349-4.

## Background

Antinuclear antibodies (ANAs) are antibodies that react against primarily self-antigens in the nucleus [1]. A positive ANA test (ANA +) is virtually a *sine qua non *for the diagnosis of systemic lupus erythematosus (SLE) since more than 95% of patients have a positive test, and the current classification criteria for SLE require a positive ANA test at a titer of ≥ 1:80 [2]. However, a positive ANA is also common in the general population and have been associated with different factors such as older age, female sex, ancestry [3], and environmental exposures [4]; approximately 12–20% are ANA + , and 2% have high titers [3].

The significance of a positive ANA in people without autoimmune disease is not known; and it is unclear whether they have altered risk of developing non-autoimmune diseases. However, ANA + individuals exhibit a unique immunological landscape [5] characterized by elevated levels of pro-inflammatory mediators and antibody production [6], as well as upregulation of some type 1 interferon (IFN) genes [7], suggesting that even in the absence of autoimmune disease, a positive ANA might alter immune regulation and affect risk of other conditions [8].

Small clinical studies suggest that ANA + can reflect increased risk for cardiovascular events [9], cancer [10], infections [11], and all cause-mortality (particularly at higher titers) [9, 12, 13]. Moreover, In vitro and animal studies have reported autoantibodies to be associated with both increased and decreased susceptibility to inflammation and models of disease [8]. For example, some autoantibodies can activate apoptosis [14] and inflammation [15], but others protect against murine polyarthritis [16], lupus-like disease [17], and kidney damage [18].

While ANAs are important biomarkers used in the diagnosis of several autoimmune diseases, the International Consensus on Antinuclear Antibody (ANA) Patterns (ICAP) has acknowledged that the term encompass antibodies directed at various cellular components and has proposed to a change in terminology to encompass 15 nuclear, 9 cytoplasmic, and 5 mitotic Hep-2 IIFA patterns [19]. However, in this study, representing samples sent for ANA testing to a hospital laboratory between 2000 and 2019, the method used for ANA testing was restricted to detect the most common anti-nuclear patterns in clinical use.

The clinical significance of ANA + , beyond its established associations with autoimmune disease, remains poorly defined largely due to an inability to study clinical outcomes in large numbers of ANA + individuals without autoimmune diseases. With the transition to the use of electronic health records (EHRs) and the development of new bioinformatic tools such as phenome-wide association studies (PheWAS)—an agnostic approach to study association between an exposure and many clinical diagnoses—it is possible to perform high-throughput phenotyping for an assessment of the associations between an exposure and a large set of phenotypes in thousands of individuals [20]. Thus, to test the hypothesis that ANA + alters the risk of some clinical disorders in the absence of an autoimmune disease, a PheWAS approach was applied to EHR data to identify the clinical associations of ANA + .

## Methods

### Study design and population

The study was approved by the Vanderbilt University Medical Center (VUMC) Institutional Review Board. We selected patients in the de-identified EHR system who had at least one ANA test ordered by clinicians as part their clinical care between October 2000 and October 2019. ANA was measured by indirect immunofluorescence (IIF) of human epithelial type 2 (HEp-2) cells, as part of usual practice by the hospital clinical laboratory using an established protocol recommended by the American College of Rheumatology (ACR) Task Force [21]. During the study period the hospital clinical laboratory performed ANA testing using anti-human IgG conjugated in Hep- 2 cells from ImmunoConcepts and Inova Diagnostics laboratory. Testing was performed following manufacturer recommendations and while manual interpretation was performed in both assays, Inova Diagnostic assay also provided automated interpretation [22]. ANA + patients were defined as those who had an ANA test with a titer ≥ 1:80; ANA negative patients (ANA-) were those who had negative ANA tests. Individuals with a titer of 1:40 and those with a reported positive test but without a titer were excluded. If multiple ANA tests were performed in the same individual, the first qualifying result was selected.

For those individuals with a positive ANA test, lab results available with 90 days of a positive ANA were extracted for following auto antibodies: anti double strand DNA (anti-dsDNA), anti-Smith (anti-Sm), anti-ribonucleoprotein (anti-RNP), anti-Ro/SSA, anti-La/SSB, anti-topoisomerase, anti-centromere, and anti-Jo1. A positive result for any of these autoantibodies were defined as being reported as “POSITIVE” or exceeding the reference values reported by the clinical lab.

The Ninth Revision and Tenth International Classification of Diseases (ICD9/ICD10) codes, which were transformed into phecodes by aggregating one or more related ICD codes into distinct diseases or traits [23], were used to differentiate patients with classical ANA-associated autoimmune disorders such as SLE, cutaneous lupus, Sjögren's syndrome, scleroderma and others (Supplementary Table S1). Patients with at least one occurrence of an ANA-associated autoimmune disease phecode constituted the autoimmune disease cohort and those with none of these phecodes were considered to not have autoimmune diseases.

### Clinical covariates

Clinical and demographic data including year of birth, sex, reported race, and length of follow-up in the EHR were extracted and used as covariates.

### Statistical analysis

#### PheWAS analysis

For everyone with an eligible ANA test, we extracted all ICD9/ICD10 codes available in the EHR and transformed them into phecodes [23, 24] were used to compare the frequency of clinical disorders between ANA + and ANA- patients. For each phenotype, cases were defined as having two or more counts of a given phecode and controls as individuals without the phecode or any closely related phecode; individuals with only one phecode were excluded [23, 24]. Only phenotypes with ≥ 200 cases were included in the analysis to improve power [25]. A multivariable logistic regression was performed adjusting for sex, year of birth, race, length of follow-up in the EHR in two analyses: a) to demonstrate proof-of-concept, the entire cohort was analyzed and b) to isolate the associations in people with and without autoimmune disease, these sub-cohorts were analyzed separately. As secondary analyses, only clinical diagnoses recorded at or after ANA testing were studied to define the temporality of the associations. In addition, stratified analyses by sex and reported race (black or white race) were performed.

Associations were expressed as odds ratio (OR) and 95% confidence interval (CI). PheWAS analyses were performed in R PheWAS package with a *P*-value ≤ 5 × 10^–5^ considered significant [26]. Demographics and disease prevalence were compared using chi-square tests and continuous variables using Wilcoxon sum rank tests.

#### Testing for confounding by frequency of ANA testing

To test for a potential directional confounding effect whereby the clinical associations with ANA + in patients without autoimmune disease could be the result of more ANA testing in these conditions, a random sample of individuals who never had an ANA test performed and who had at least one ICD9/ICD10 code in the EHR, and no autoimmune disease was selected. These individuals were frequency matched to those in the ANA tested sub-cohort without autoimmune disease for year of birth, sex, reported race, and length of follow-up in the EHR.

The log odds (or beta estimate) of being tested for each clinical diagnosis was estimated by comparing the frequency of each clinical diagnosis in the group who had never been tested for ANA and the study sub-cohort without autoimmune disease who had been tested for ANA using a PheWAS approach. Spearman’s test was used to assess if there was a correlation between the log odds of being tested for ANA and of being ANA + for clinical diagnoses with ≥ 200 cases. A *P*-value ≤ 0.05 was considered significant.

## Results

Characteristics of individuals with an ANA test: As of October 2019, there were 76,201 individuals with an ANA result in their EHR. After excluding individuals with an ANA titer of 1:40 or a positive ANA test result without titer information (*n* = 4,713), those with conflicting or missing information for sex (*n* = 978) and for length of follow-up (*n* = 61), and those with no ICD9/ICD10 codes in their EHR (*n* = 406), 70,043 individuals remained in the study cohort. Of these, 29% (20,497) had a diagnosis of an ANA-associated autoimmune disease. Table 1 shows the characteristics of the study cohort: 38% (26,579) were ANA + and were 62% (43,464) ANA-. Most demographic characteristics were similar between both groups, but a higher proportion of the ANA + group were women (78.1% vs. 62.8%).

**Table 1:** Characteristics of individuals with an antinuclear antibody test

Characteristics	ANA tested cohort (*n* = 70,043)	ANA positive (*n* = 26,579)	ANA negative (*n* = 43,464)
Female (%)	48,051 (68.6%)	20,755 (78.1%)	27,296 (62.8%)
White race (%)^a^	52,475 (74.9%)	19,857 (74.7%)	32,618 (75%)
Year of birth	1963 [1951, 1977]	1962 [1950, 1976]	1963 [1952, 1978]
Age at testing (years)	48.4 [34.5, 59.7]	49.9 [35.9, 61.3]	47.5 [33.7, 58.6]
Length of FU (years)	7.09 [1.88, 13.10]	7.23 [1.92, 13.04]	7.01 [1.85, 13.14]

*ANA* Antinuclear antibodies, *FU* Follow-up

^a^Race recorded as white in the electronic heath record (EHR). Data are shown as counts (percentage) for categorical variables and median [interquartile range] for continuous variables

Characteristics of individuals with and without autoimmune diseases: The demographic and clinical characteristics of individuals in the study cohort with and without autoimmune disease differed (Table 2). In individuals with autoimmune disease (20,497), 53.3% (10,931) were ANA + with titers of 1:80 in 14.4%, 1:160 in 66.3%, 1:320 in 8.6%, and ≥ 1:640 in 10.6%. ANA patterns were reported in 42.5% of those with a positive test and a homogeneous pattern (62.9%) was the most common pattern reported, followed by speckled (26.4%), centromere (4.2%), nucleolar (3.9%), and atypical (2.7%). Additional autoantibody results were reported in 72.6% (7,937) of patients with autoimmune disease and a positive ANA, and 20.4% (1622) a positive test for at least one of these autoantibodies: anti-dsDNA 9.8% (641 of 6527 tested), anti-Sm 2.1% (146 of 6843 tested), anti-RNP 5.1% (337 of 6667 tested), anti-Ro/SSA 8.7% (613 of 7021 tested), anti-La/SSB 6.2% (422 of 6815 tested), anti-Scl70 2.1% (143 of 6678 tested), and anti-Jo1 8.1% (13 of 160 tested).

**Table 2:** Characteristics of individuals with and without an ANA-related autoimmune disease

Characteristics	With AD (*n* = 20,497)	ANA + (*n* = 10, 931)	ANA- (*n* = 9,566)	Without AD (*n* = 49,546)	ANA + (*n* = 15,648)	ANA- (*n* = 33,898)
Female (%)	15,733 (76.8%)	9,167 (83.9%)	6566 (68.6%)	32,612 (65.3%)	11,588 (74.1%)	20,730 (61.2%)
White race (%)^a^	15,957 (77.9%)	8,305 (76.0%)	7652 (80.0%)	36,518 (73.7%)	11,552 (73.8%)	24,966 (73.7%)
Year of birth	1962 [1951, 1975]	1962 [1950, 1976]	1961 [1951, 1975]	1963 [1951, 1978]	1962 [1950, 1976]	1964 [1952, 1978]
Age at testing (years)	49.4 [36.2, 60.2]	49.5 [35.7, 60.7]	49.2 [36.7, 59.5]	47.9 [33.8, 59.5]	50.2 [35.9, 61.8]	46.9 [32.9, 58.3]
Length of FU (years)	8.7 [3.4, 14.1]	8.6 [3.2, 13.9]	8.9 [3.6, 14.4]	6.4 [1.3, 12.6]	6.2 [1.1, 12.4]	6.4 [1.4, 12.7]

*ANA* Antinuclear antibodies, *ANA* + ANA positive, *ANA-* ANA negative, *AD* Autoimmune disease, *FU* Follow-up

^a^Race recorded as white in the electronic heath record (EHR). Data is shows as counts (percentage) for categorical variables and median [interquartile range] for continuous variables

In patients without autoimmune disease (49,952), 31.7% (15,648) were ANA + with a titer of 1:80 in 22.5%, 1:160 in 59.7%, 1:320 in 12.2% and ≥ 1:640 in 5.6%. An ANA pattern was reported in 43.7% (6,839) of the ANA + individuals, and 65.9% had a homogeneous pattern, followed by speckled in 26.7%, nucleolar in 4.4%, centromere in 1.6%, and atypical in 1.4%. ANA + was more common in women in both groups.

Autoantibody results were available in 55.0% (8758) of individuals without an autoimmune disease and positive ANA; and in 5.3% (461) a positive for at least one of these autoantibodies: anti-dsDNA 1.1%, (77 of 6809 tested), anti-Sm 0.4% (31 of 7440 tested), anti-RNP 1.1% (84 of 7334 tested), anti-Ro/SSA 1.8% (139 of 7940 tested), anti-La/SSB 1.8% (136 of 7769 tested), anti-Scl70 1.2% (85 of 7310 tested), and anti-Jo1 0.8% (1 of 121 tested).

**Fig. 1:**
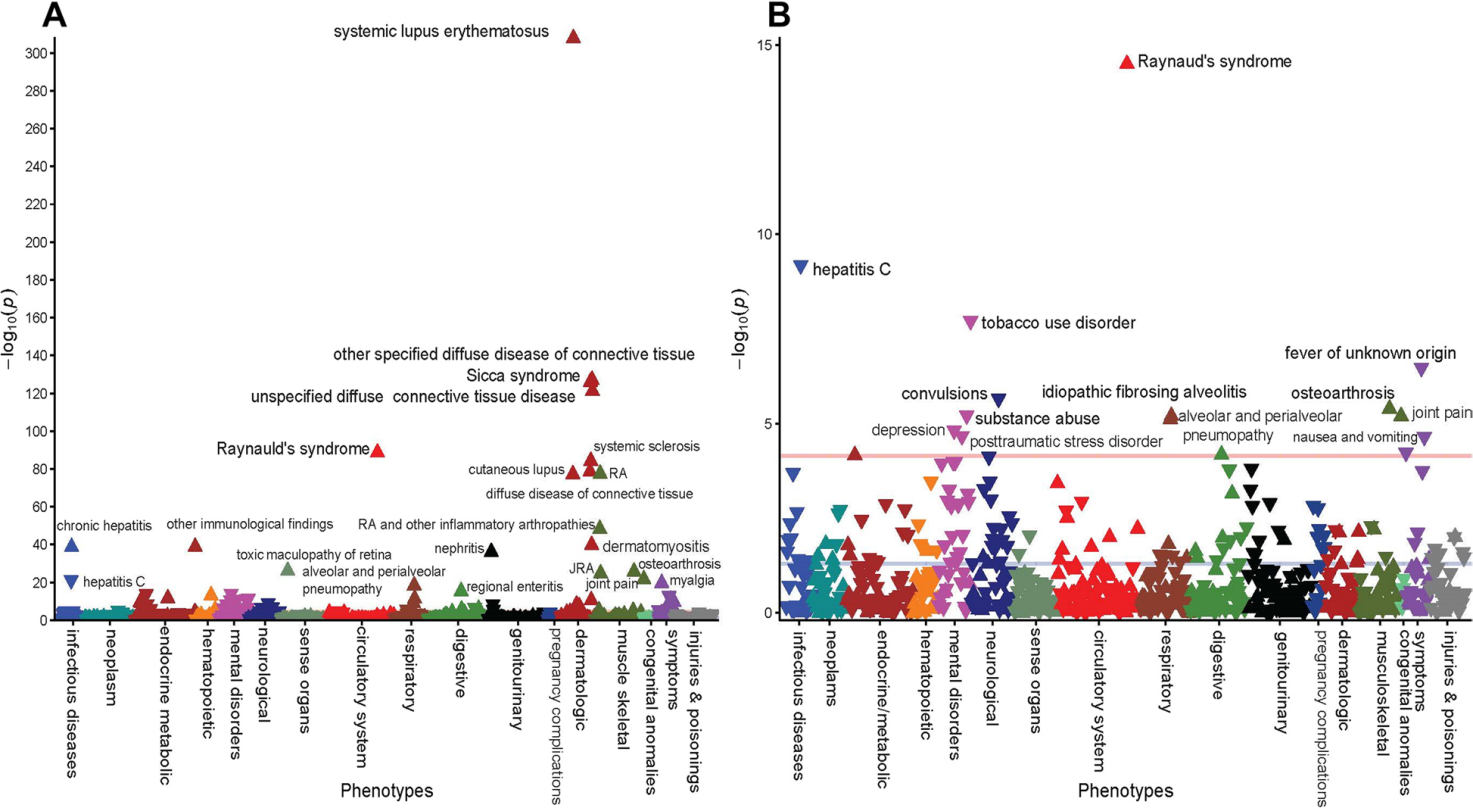
Clinical diagnoses associated with positive antinuclear antibodies (titer ≥ 1:80) in (**A**) all individuals tested and (**B**) in individuals without and autoimmune diseases. ▲ and ▼represent increased (OR > 1) and decreased (OR < 1) risk, respectively. Red horizontal line represents *P* ≤ 5 × 10^–5^. Twenty-two diagnoses with the most significant *P*-value association of the 88 significant associations are labeled in the whole cohort (**A**) and all 13 significant associations are labeled in those without an autoimmune disease (**B**)

PheWAS for ANA + versus ANA- in the study cohort: In the PheWAS that included all study individuals (ANA + and ANA-, *n* = 70,043), 88 clinical diagnoses were significantly (*P* ≤ 5 × 10^–5^) associated with ANA + (Fig. 1A). Known clinical associations with ANA + (SLE, sicca syndrome, UCTD, systemic sclerosis, RA-related disorders, etc.) were among the top associations (all *P* ≤ 1 × 10^–20^, Table 3, Supplementary Table S2). ANA + was also associated with decreased prevalence of several non- autoimmune disorders including viral hepatitis C (OR = 0.66, *P* = 6.1 × 10^–22^), hypertension (OR = 0.9, *P* = 1.7 × 10^–5^), abdominal pain (OR = 0.9, *P* = 1.6 × 10^–13^), convulsions (OR = 0.8, P = 9.1 × 10^–10^), acute renal failure (OR = 0.9, *P* = 3.5 × 10^–9^), type 2 diabetes (OR = 0.8, *P* = 1.5 × 10^–14^) and several of its complications (OR < 0.9, *P* ≤ 5.1 × 10^–6^), and several psychiatric disorders (mood disorders, bipolar, post-traumatic stress disorder, altered mental status, among others), and substance abuse disorders (e.g., tobacco disorder, substance abuse, alcoholism; Supplementary Table S2). Excluding diagnoses that were recorded before ANA testing, yield similar results (Supplementary Table S3).

**Table 3:** Significant clinical associations with positive antinuclear antibodies (titer ≥ 1:80) in all patients tested and the findings for those diagnoses in those with and without autoimmune disease

**Phecode**	**Clinical diagnosis**	**All ANA tested**^b^		**With AD**^b^		**Without AD**^b^	
		**OR**	***P*-value**	**OR**	***P*-value**	**OR**	***P*-value**
695.42	Systemic lupus erythematosus^a^	9.1	0.0E + 00	5.4	7.8E-202	-	-
709.6	Other specified diffuse diseases of connective tissue^a^	7.4	7.5E-128	5.4	8.9E-86	-	-
709.2	Sicca syndrome^a^	4.8	9.7E-127	3.6	8.6E-77	-	-
709.7	Unspecified diffuse connective tissue disease^a^	8.0	5.9E-122	5.6	4.2E-80	-	-
443.1	Raynaud's syndrome^a^	3.4	2.2E-89	3.4	4.5E-42	2.1	3.2E-15^c^
709.3	Systemic sclerosis^a^	11.9	5.7E-85	8.8	1.9E-64	-	-
709	Diffuse diseases of connective tissue^a^	19.5	2.3E-79	13.4	2.1E-59	-	-
695.41	Cutaneous lupus erythematosus^a^	5.3	6.1E-78	3.2	4.5E-37	-	-
709.5	Dermatomyositis^a^	5.1	1.5E-40	3.6	1.0E-25	-	-
279.7	Other immunological findings^a^	5.4	1.7E-39	3.6	9.7E-14	-	-
580.31	Nephritis & nephropathy in diseases classified elsewhere	2.6	7.3E-37	2.7	2.7E-19	1.34	0.76
362.5	Toxic maculopathy of retina	5.2	7.6E-27	3.2	2.7E-12	-	-
740.2	Osteoarthrosis, generalized	1.4	1.9E-26	1.1	0.32	1.4	4.0E-06^c^
745	Pain in joint	1.2	2.4E-22	1.4	0.29	1.1	6.3E-06^c^
70.3	Viral hepatitis C	0.7	6.1E-22	0.5	4.8E-23	0.7	6.5E-10^c^
504	Other alveolar and parietoalveolar pneumonopathy	1.7	4.4E-19	1.6	5.4E-07	1.4	7.7E-06^c^
296.2	Depression	0.8	1.0E-14	0.7	1.9E-14	0.8	1.5E-05^c^
250.2	Type 2 diabetes	0.8	1.5E-14	0.7	4.0E-23	0.9	3.5E-03
286.81	Primary hypercoagulable state	1.8	6.8E-14	1.8	1.4E-06	1.7	0.02
785	Abdominal pain	0.9	1.6E-13	0.7	7.0E-26	0.91	1.8E-04
318	Tobacco use disorder	0.8	4.2E-12	0.8	1.0E-07	0.8	1.9E-08^c^
504.1	Idiopathic fibrosing alveolitis	1.6	4.1E-12	1.6	1.75E-04	1.5	6.3E-06^c^
316	Substance addiction and disorders	0.7	6.7E-12	0.6	3.6E-09	0.8	6.0E-06^c^
300.1	Anxiety disorder	0.8	1.4E-11	0.7	2.5E-14	0.9	1.4E-03
296.22	Major depressive disorder	0.8	2.7E-11	0.7	6.3E-13	0.9	1.0E-04
296.1	Bipolar	0.7	2.8E-11	0.6	1.9E-07	0.8	1.0E-04
709.4	Polymyositis^a^	2.6	3.1E-11	1.9	1.0E-05	-	-
300.9	Posttraumatic stress disorder	0.7	1.5E-10	0.6	1.6E-06	0.7	2.1E-05^c^
345.3	Convulsions	0.8	9.1E-10	0.7	9.8E-06	0.8	2.2E-06^c^
585.1	Acute renal failure	0.9	3.5E-09	0.7	4.5E-18	0.9	1.5E-04
250.22	Type 2 diabetes with renal manifestations	0.8	3.6E-09	0.6	6.2E-15	0.9	0.09
292.4	Altered mental status	0.8	5.2E-09	0.6	3.1E-13	0.9	1.1E-03
250.1	Type 1 diabetes	0.7	1.5E-08	0.5	3.5E-14	0.9	0.06
296	Mood disorders	0.8	9.3E-08	0.6	1.4E-10	0.8	9.3E-03
348.8	Encephalopathy, not elsewhere classified	0.8	1.9E-07	0.6	6.2E-11	0.8	5.9E-03
300.11	Generalized anxiety disorder	0.7	2.1E-07	0.7	3.3E-05	0.8	1.3E-03
571.8	Liver abscess and sequelae of chronic liver disease	0.8	3.0E-07	0.5	4.6E-22	0.9	0.02
291.8	Alteration of consciousness	0.8	8.6E-07	0.7	1.0E-05	0.8	1.1E-04
783	Fever of unknown origin	0.9	1.3E-06	0.7	7.1E-14	0.8	3.3E-07^c^
250.6	Polyneuropathy in diabetes	0.8	1.9E-06	0.6	2.5E-08	0.9	0.04
555.2	Ulcerative colitis^a^	1.4	2.7E-06	0.7	2.2E-06	-	-
495	Asthma	0.9	2.9E-06	0.7	1.2E-09	0.9	0.03
585.2	Renal failure NOS	0.8	3.3E-06	0.6	3.7E-08	0.8	1.5E-03
760	Back pain	0.9	4.5E-06	0.8	5.8E-08	0.9	5.8E-05
250.24	Type 2 diabetes with neurological manifestations	0.8	5.0E-06	0.6	5.5E-16	1.0	0.66
250.23	Type 2 diabetes with ophthalmic manifestations	0.7	5.1E-06	0.5	2.0E-09	0.9	0.21
789	Nausea and vomiting	0.9	6.6E-06	0.8	2.2E-12	0.9	2.2E-05^c^
401.1	Essential hypertension	0.9	1.7E-05	0.8	1.7E-06	0.9	2.0E-03
250	Diabetes mellitus	0.8	2.5E-05	0.6	3.1E-09	0.9	0.08
496	Chronic airway obstruction	0.8	2.7E-05	0.7	1.2E-08	0.9	0.06

Data are shown for the 50 of the 83 significant (*P* < 5.0E-05) associations in the whole cohort. All significant associations for the cohorts are shown in Supplement Tables (S2, S3 and S4)

*ANA* Antinuclear antibody, *AD* ANA-related autoimmune disease, *OR* Odds ratio. Data is not shown for those diagnoses with < 200 cases

^a^Autoimmune disorders excluded from the cohort without autoimmune disease

^b^Adjusting for sex, year of birth, race, length of follow-up in the HER

^c^Significant associations in patients without autoimmune diseases

PheWAS for ANA + versus ANA- in the sub-cohort with autoimmune diseases: When individuals with autoimmune diseases were analyzed separately, 136 clinical diagnoses were significantly associated with ANA + (*P* ≤ 5 × 10^–5^, Supplementary Table S4). The top associations included autoimmune disorders (Table 3). Most of the significant associations in the autoimmune disease sub-cohort have the same direction observed in the entire cohort (Table 3), including the those diagnoses with inverse associations (e.g., hepatitis C, diabetes, mood disorders, Type 2 diabetes, hypertension, etc.) (Supplementary Table S4, Table 3).

PheWAS for ANA + versus ANA- in the sub-cohort without autoimmune disease: In the sub-cohort without autoimmune disease (*n* = 49,952) there were 13 clinical diagnoses significantly associated with ANA + (*P* ≤ 5 × 10^–5^, Table 3, Fig. 1B), with Raynaud’s syndrome as the top association (OR = 2.1, *P* = 3.2 × 10^–15^), followed by viral hepatitis C (OR = 0.7, *P* = 6.5 × 10^–10^), tobacco use disorder (OR = 0.8, *P* = 1.9 × 10^–8^), fever (OR = 0.8, *P* = 3.4 × 10^–7^), convulsions (OR = 0.8, *P* = 2.2 × 10^–6^), osteoarthrosis (OR = 1.3, *P* = 4.0 × 10^–6^), substance addiction and disorders (OR = 0.8, *P* = 6.0 × 10^–6^), idiopathic fibrosing alveolitis (OR = 1.5, *P* = 6.3 × 10^–6^), join pain (OR = 1.1, *P* = 6.3 × 10^–6^), alveolar pneumopathies (OR 1.4, *P* = 7.7 × 10^–6^), and depression (OR 0.8, *P* = 1.5 × 10^–5^) (Supplementary Table S5). For these phenotypes, results were similar in direction and statistical significance in the other two cohorts (Table 3); as well as in the analysis that include only clinical diagnoses after ANA testing (Supplementary Table S6).

When females and males in the sub-cohort without autoimmune disease were analyzed separately, eighteen clinical diagnoses were significantly associated with ANA + in females (*n* = 32,318, Supplementary Table S7) and 2 in males (*n* = 17,228, Supplementary Table S8). In the analysis stratified by race, seven clinical diagnoses were significantly associated with ANA + in white individuals (*n* = 36,518, Supplementary Table S9) while only one (osteoarthrosis) in black individuals (*n* = 5,493, Supplementary Table S10). Most of the significant associations in the stratified analyses (by sex and race) were also seen in the previous cohorts and had the same direction of effect.

When cases of osteoarthrosis (OA) were separated by location based on ICD codes, ANA+ was associated with OA in hands (OR = 1.2, *P* = 1.9 × 10^–6^), wrist (OR = 1.3, *P* = 0.03), knees (OR = 1.1, *P* = 0.002), and hips (OR = 1.1 *P* = 0.004). The associations were no longer significant when we adjusted for clinical covariates (sex, year of birth, race, length of follow-up in the EHR, all *P*-values > 0.2). Positive ANA was associated with increased risk of OA in multiple sites in the univariate (OR = 1.5, *P* = 2 × 10^–6^) and fully adjusted model (OR = 1.3, *P* = 8 × 10^–14^).

Confounding by frequency of ANA testing: We compared the sub-cohort of individuals without any autoimmune disorder with a random sample frequency matched of 45,473 individuals from the EHR using PheWAS and correlated the regression coefficients of being tested for ANA with the coefficients of a having a positive ANA test for phecodes with ≥ 200 cases (704 clinical diagnoses). There was no significant correlation between the coefficients of being tested and being ANA + (Spearman correlatio*n* = 0.001, *P* = 0.796, Supplementary Figure S1).

## Discussion

A positive ANA test was strongly associated with several autoimmune diseases or their complications in the entire cohort and the sub-cohort with autoimmune diseases, an expected finding and one that supports the approach used. Additionally, ANA + in individuals without autoimmune diseases was associated with increased risk of five clinical diagnoses and decreased risk of eight.

In the entire cohort, as well as in the sub-cohort of individuals with autoimmune disorders, ANA + was strongly associated with increased risk of autoimmune disorders for which a positive test is characteristic (e.g. SLE [8], systemic sclerosis [27], and UCTD [28]) and those for which it is a common feature (e.g. Sjogren’s, myositis) [29]. ANA + was also associated with increased risk for symptoms/complications related to these autoimmune diseases or their treatment (e.g., nephritis, myalgia, toxic maculopathy of retina-related to hydroxychloroquine use). In addition, ANA + showed a significant inverse association with several clinical diagnoses (48 in the entire cohort and 121 in the sub-cohort with autoimmune disorders), most of which were non- autoimmune disorders. Similar results were observed when only clinical diagnoses that were recorded after ANA testing were analyzed.

Some (but not all) previous epidemiologic studies have found that ANA + was associated with increased risk of various cardiovascular events [9, 12, 13], cancers [10], and all-cause mortality [12]. We did not find an association between ANA + and any cardiovascular phenotype or cancer and were unable to test the relationship with overall mortality.

Novel findings were that in the cohort of individuals without autoimmune diseases, ANA + increased the risk for five phenotypes and decreased risk for eight. However, there was little evidence of immune dysregulation as evidenced by no increase in infections, cancer, or renal failure, which is consistent with previous findings where ANA + individuals exhibited a unique immune suppressive signature compared to ANA- individuals [5]. This unique signature was characterized by reduced number of T-cells, reduced levels of proinflammatory soluble mediators in plasma, dysregulated T-cell signaling, and decreased expression of interferon-inducible and HLA class I genes, which may prevent the onset of clinical autoimmunity [5].

Concordant with previous studies, ANA + was associated with increased the risk of Raynaud’s syndrome [30] and disorders related to idiopathic fibrosing alveolitis [31]. The risk of osteoarthritis and related symptoms (joint pain) was increased in ANA + patients, a consistent finding across the cohorts. Interestingly, a previous small clinical study found that ANA + was associated with more severe OA [32].

While OA is thought to be a seronegative disease; there are several possible explanations for our findings. It is possible that some of the patients diagnosed with OA might have an undiagnosed autoimmune condition. Another possibility is that OA may increase autoantibodies production. Synovitis, which is a characteristic of OA, has been associated with post translational modifications of proteins [33], by citrullination, oxidation, glycation or carbamylation. These protein modifications are an important source of antigenicity for antibody production and also play an important role in disease [34, 35]. Circulating autoantibodies to native collagen proteins and to carbamylated proteins have been reported to be more common in patients with OA compared to healthy controls [36]. but their clinical significance of these autoantibodies remain unknown.

In all cohorts there was an inverse association between ANA + and hepatitis C, mood disorders, tobacco use disorders, substance use disorders, and convulsions. Although the reported prevalence of ANA + among patient with hepatitis C infection ranges from 3 to 63% [37], the inverse association observed between ANA + and hepatitis C was unexpected. The inverse associations between ANA + and mood disorders are also novel. Previous small clinical studies have shown mixed results [38], but a recent study of 368 patients with mood disorders and 283 controls found no differences in prevalence of ANA + [39]. However, in a representative sample from the National Health and Nutrition Examination Survey (NHANES) ANA + was inversely associated with recent use of some psychotherapeutic drugs (OR = 0.64, 95%CI = 0.43, 0.95), including antidepressants (OR = 0.64, 95%CI = 0.42, 0.97), particularly serotonin reuptake inhibitors (OR = 0.65; 95%CI = 0.42, 0.98) [40].

The inverse association between tobacco use disorder and ANA + was consistent among all cohorts. Although smoking is associated with increased risk of several inflammatory diseases [41] and has been associated with high levels of anticitrullinated cyclic peptide/protein antibody (ACPA) in patients with RA [42] with similar trend in the general population [43]; an epidemiological study previously reported that active smoking was weakly associated with lower ANA levels [44]. In keeping with this observation, several studies suggest that smoking has a suppressive effect on autoimmunity [45] (with impairment of the antibody-forming cell response and lower levels of several immunoglobulins) [46], which recovers after smoking cessation [47]. In animal models, nicotine dampens the inflammatory response [48], and administration of nicotine reduced inflammation [49] in patients with ulcerative colitis [50], and lupus [48].

The inverse association between ANA + and substance use has not been reported previously. However, clinical and experimental animal studies have shown that exposure to drugs can lead to immune dysregulation and impair antibody production [51, 52]. Whether the inverse association found with ANA + with substance use reflects the direct effect of the used drugs or related factors [53] on the immune system is not known.

Likewise, the inverse association observed between ANA + and seizures is novel. Small clinical studies have reported conflicting results about the prevalence of ANA + in individuals with epilepsy compared to healthy controls [54] as well as the effect of antiepileptic drug on ANA positivity [54, 55].

The inverse association between ANA + and hypertension and type 2 diabetes-related phenotypes was consistent in all cohorts. The mechanism for these inverse associations is unclear, but the study in NHANES reported an inverse association between ANA + and thiazide diuretics and sulfonylurea antidiabetic drugs [40].

As expected, ANA + was associated with nephritis [56] in the entire cohort and in the sub-cohort of autoimmune disorders; however, there was an unexpected inverse association between ANA + and renal failure (acute and chronic) in all cohorts (with nominal associations in the sub-cohort without autoimmune disease, *P*< 0.05). The concept that some autoantibodies need not be harmful but can be protective has been suggested and is supported by several lines of evidence [8], including the immune suppressive profile described in ANA + individuals [5].

Certain ANAs, like antibodies against a nuclear DNA-binding protein (HMGB1), decreased albuminuria, complement deposition, and neutrophil recruitment in a murine lupus model [18]. Other “natural autoantibodies” that are largely of the IgM class and bind many self- and non-self-antigens prevented proteinuria and reduced kidney immune complex deposition in a murine lupus model [57]. The specificity of ANAs occurring in healthy people is poorly characterized [1] and, as suggested by Silverman [58], a positive ANA test in some settings could represent the tip of the iceberg of circulating natural autoantibodies.

Additionally, ANAs may alter disease manifestations. The phenotype of seropositive patients with RA who were also ANA + differed from those who were ANA- in that it took longer for them to fulfill RA criteria and require treatment with disease-modifying antirheumatic drugs [59]. We were unable to examine the course of diseases such as RA within the design of our study.

The limitations of this study include:

- ANA tests are not performed randomly in clinical practice but rather are requested based on clinical suspicions. However, the indication for ANA testing can usually only be inferred from EHRs and would require manual review of thousands of records for hundreds of diagnoses, something clearly not feasible. Nevertheless, we considered the possibility that increased ANA testing for patients with a particular non-autoimmune diagnosis might confound the associations between a positive test and that diagnosis. However, there was no significant correlation between the frequency of ANA testing and the prevalence of ANA + across a range of diseases. Ideally, for each phenotype, positive and negative ANA test frequencies should be compared in a random sample of patients affected with the disease and a random sample of age, race, and sex matched controls without the phenotype. It is methodologically not feasible to perform such studies on a large scale and match groups across a wide range of phenotype-specific covariates. Instead, we used a broad approach and sought to minimize confounding by excluding individuals with autoimmune disorders and adjust for key covariates.
- While ANA testing followed the position statement of the American College of Rheumatology, changes in personnel or laboratory practices could have affected assays, factors inherent to real-world data. However, the expected steady and strong associations between ANA and autoimmune disease suggest the ANA assay was robust. Nevertheless, the ICAP committee has being working on the nomenclature and definitions of HEp-2 IIFA patterns since 2014, and in 2019 the executive ICAP members published a consensus paper regarding the clinical relevance of 29 distinct Hep-2 IFFA patterns to support clinical decisions. The use of such recommended nomenclature in future prospective studies would harmonize ANA testing and reporting allowing future systematic reviews to further fine-tune current consensus based on expert opinions [19].
- Anti-DFS70 antibody is an autoantibody that reacts against a nuclear chromatin-associated protein that can be detected in standard IIF ANA testing [60]. While it is possible that some of positive ANA results are due to this antibody, we would have expected to find significant associations with some of the conditions associated with the presence of anti-DFS70 antibodies—atopic dermatitis, eye conditions, and prostate cancer [60] in the sub cohort of individuals without autoimmune disorders. Instead, we found positive association with diseases known to have an autoimmune basis like Raynaud’s disease and idiopathic fibrotic alveolitis.
- Study findings cannot be generalized to the general population since the study population derived from a tertiary-care hospital.
- Billing codes were used to assemble phenotypes, and the quality of the case–control definition could vary across phenotypes and lead to some misclassification [61]. While validation of hundreds of clinical diagnoses in thousands of individuals is not possible in studies using extensive real-world clinical data, the strong and consistent associations observed between ANA and autoimmune disorders for which a positive ANA is characteristic (e.g. SLE) in different analyses supports the robustness of phenotype assignments.
- It is possible that some ANA + individuals develop an autoimmune disease later in life [62], which could have biased our results. Our approach of using all diagnoses in the EHR (i.e., diagnoses occurring both before and after the positive ANA test) mitigated this problem since autoimmune conditions that developed later in follow up would have categorized the patient correctly.
- The production of ANA can be induced by some medications, and we could not specifically define such drug-induced positive ANAs. However, we did not find significant positive associations between disorders that are commonly treated with such drugs (e.g., arrythmias, tuberculosis, acne, etc.) and ANA + in individuals without autoimmune diseases.
- The study design only detected associations and not mechanisms or causality; also, confounding or reverse causation cannot be ruled out.

Although our approach has limitations due to the nature of the data, traditional epidemiologic studies to define the clinical impact of a positive ANA test are not feasible. The major strength of the study is that we leveraged real-world clinical data from EHRs to perform high-throughput screening for clinical associations with a positive ANA in a population of more than 70,000 individuals from a specialized medical center, which yielded consistent results in all analyses.

In conclusion, this large EHR study of patients tested for ANA confirmed the association of ANA + with several autoimmune disorders, and in patients without autoimmune disease, ANA + was associated with an increased risk of Raynaud’s and idiopathic fibrosing alveolitis related disorders and decreased prevalence of several non- autoimmune diseases.

### Supplementary Information

**Additional file 1.**

**Additional file 2.**

## Data Availability

The datasets generated and/or analysed during the current study are available from the correspondig author on reasonable request. Phecode maps and R code for the Phewas analysis are openly available at https://phewascatalog.org/.

## Abbreviations

ANA: Antinuclear antibodies
EHR: Electronic health record
VUMC: Vanderbilt University Medical Center
SLE: Systemic lupus erythematosus
IFN: Interferon
PheWAS: Phenome-wide assocation study
IIF: Indirect immunofluorescence
HEp-2: Human epithelial type 2
ACR: American College of Rheumatology
ICD: International classification of Diseases
OR: Odds ratio
CI: Confidence interval
UCTD: Undifferentiated connective tissue disease
RA: Rheumatodi arthritis
NHANES: National Health and Nutrition Examiniation Survey
HMGB1: High mobility group box 1
Ig: Immunoglobulin
DFS70: Dense fine speckled, 70 kDa molecular weight
