# Supplementary figures and images for: Clinical diagnoses associated with a positive antinuclear antibody test in patients with and without autoimmune disease
Source: BMC Rheumatol. 2023 Aug 7;7:24. doi: 10.1186/s41927-023-00349-4 (PMC10405518; doi:10.1186/s41927-023-00349-4)

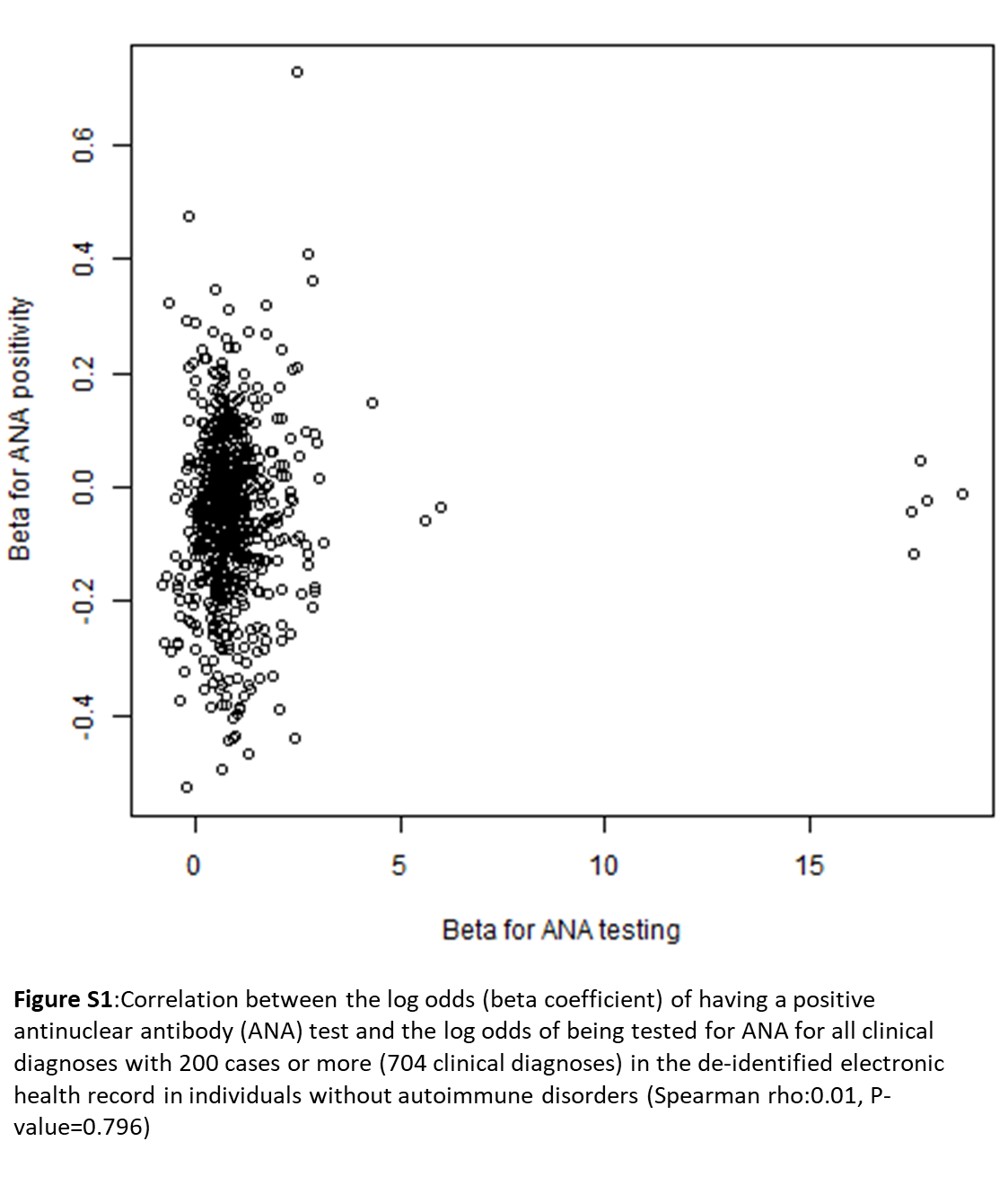

Supplement: Supplementary file 1 — Additional file 1. [file 41927_2023_349_MOESM1_ESM.docx]
